# Alzheimer’s disease: Pathophysiology and dental pulp stem cells therapeutic prospects

**DOI:** 10.3389/fcell.2022.999024

**Published:** 2022-09-15

**Authors:** Wei Xiong, Ye Liu, Heng Zhou, Shuili Jing, Yan He, Qingsong Ye

**Affiliations:** ^1^ Center of Regenerative Medicine, Renmin Hospital of Wuhan University, Wuhan University, Wuhan, China; ^2^ Institute of Regenerative and Translational Medicine, Tianyou Hospital, Wuhan University of Science and Technology, Wuhan, China

**Keywords:** Alzheimer’s disease, DPSCs, stem cell therapy, neuroregeneration, regeneration medicine

## Abstract

Alzheimer’s disease (AD) is a destructive neurodegenerative disease with the progressive dysfunction, structural disorders and decreased numbers of neurons in the brain, which leads to long-term memory impairment and cognitive decline. There is a growing consensus that the development of AD has several molecular mechanisms similar to those of other neurodegenerative diseases, including excessive accumulation of misfolded proteins and neurotoxic substances produced by hyperactivated microglia. Nonetheless, there is currently a lack of effective drug candidates to delay or prevent the progression of the disease. Based on the excellent regenerative and reparative capabilities of stem cells, the application of them to repair or replace injured neurons carries enormous promise. Dental pulp stem cells (DPSCs), originated from ectomesenchyme of the cranial neural crest, hold a remarkable potential for neuronal differentiation, and additionally express a variety of neurotrophic factors that contribute to a protective effect on injured neuronal cells. Notably, DPSCs can also express immunoregulatory factors to control neuroinflammation and potentiate the regeneration and recovery of injured neurons. These extraordinary features along with accessibility make DPSCs an attractive source of postnatal stem cells for the regeneration of neurons or protection of existing neural circuitry in the neurodegenerative diseases. The present reviews the latest research advance in the pathophysiology of AD and elaborate the neurodifferentiation and neuroprotective properties of DPSCs as well as their application prospects in AD.

## Graphical Abstract

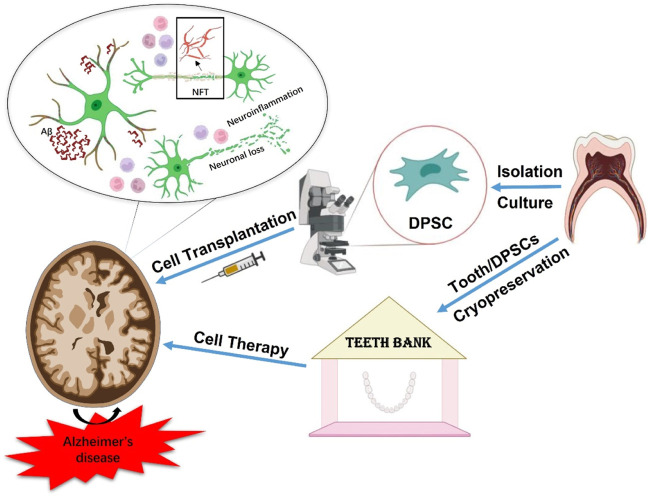

## 1 Introduction

Alzheimer’s disease (AD) is highly disabling, and debilitating disorder characterized by progressive loss of functional neurons and cognitive impairment. As the most common cause of dementia, the incidence of AD is accounting for 50%–75% of all dementias. According to the World Alzheimer’s Disease Investigation Report showed that dementia is currently the seventh leading cause of mortality globally and is becoming a worldwide epidemic, with 152 million cases expected by 2050 (Alzheimer’s Disease International, 2021). Mortality and home care costs for AD have been further aggravated with the pandemic and normalization of the COVID-19, which undoubtedly poses a great challenge to national health care (Alzheimer’s Disease Facts and Figures, 2021). Intensive research work on AD has shown that age is one of the most predominant risk factors for AD and aging makes us highly susceptible to age-related diseases such as neurodegenerative diseases (Mertens et al., 2018). Thus, while mutations in amyloid precursor protein (APP), presenilin 1 (PS1) and presenilin (PS2) cause a rare familial AD (FAD), the vast majority of AD is sporadic. Current studies show that approximately 70% of sporadic AD is attributable to genetic and environmental factors, with three variants of the ApoE gene are the strongest risk causative genes (Lane et al., 2018). Therefore, this leads to the uncertainty of the aetiology and the variety of pathology in AD.

Up to now, the academic research on AD mainly focuses on four core neuropathological hallmarks. Firstly, one of the features of AD is the occurrence and deposition of amyloid plaques in the neural extracellular matrix of brain. This is mainly due to the cleavage and degradation of APP protein by β- and γ-secretases resulting in the extracellular accumulation of Aβ protein fragments. Secondly, hyperphosphorylated tau, an intracellular microtubule-associated protein (Grundke-Iqbal et al., 1986), causes microtubule collapse and neurofibrillary tangles in nerve cells. Thirdly, Hyperactivated microglia, neuroinflammation and oxidative stress also belong to the pathological features of AD. The last core feature is the damage and loss of numerous neurons and synapses, which may be a reason of early cognitive decline in AD patients (Delbeuck et al., 2003). As the disease progresses, the hippocampus and other brain regions eventually atrophy under a mixed action of various neuropathological lesions, thus greatly impairing the quality of life and longevity of AD patients.

Given the great proliferative capacity and differentiation potential of exogenous stem cells, therapy based on them has been well substantiated to ameliorate impaired tissue and microenvironment disorders via direct cell-cell interactions or paracrine action (Sivandzade and Cucullo, 2021). And the stem cell therapy for AD has made good progress in animal models (Jia et al., 2020; Santamaria et al., 2021). This seems to suggest that the clinical application of stem cell transplantation for AD is within reach. Recently, especially in the context of neurodegenerative diseases, DPSCs have drawn much attention as mesenchymal stem cells (MSCs) with a wide range of sources and low immunogenicity. DPSCs originate from migrating the cranial neural crest lineage and dwell in the perivascular niche of dental pulp. It possess the general properties of MSCs, such as the potential for self-renewal and multi-lineage differentiation, which have been confirmed to differentiate into osteoblasts, adipocytes, chondrocytes and neuroectodermal cells (Ledesma-Martinez et al., 2016). Attributing to the peculiar neural crest origin, DPSCs could highly present neural biomarkers such as nestin, β-tubulin III, glial fibrillary acidic protein (GFAP) and microtubule-associated protein-2 (MAP-2) (Kiraly et al., 2009; Sakai et al., 2012). Besides, DPSCs may differentiate into functionally neuron-like cells and oligodendrocyte-like cells under neuronal differentiation conditions, which can repair the cellular damage caused by central nervous system diseases (Arthur et al., 2008; Askari et al., 2014). DPSCs also secrete brain-derived neurotrophic factor (BDNF), glial cell-derived neurotrophic factor (GDNF) and Neurotrophin-3 (NT-3) to protect and nourish neurons in the damaged nervous microenvironment (Luo et al., 2018a).

Therefore, the goal of improvement in neurological function may involve a complex process of neurogenesis, neuroprotection, and immunomodulation of inflammation. A better understanding of the underlying neuropathogenesis and molecular mechanisms may contribute to select more appropriate stem cell types and more targeted treatment strategies, which can help to delay or even reverse AD. Here, this review has outlined and discussed the cutting-edge AD research progress, as well as the outstanding advantages of DPSCs in AD treatment, which provides valuable insights into the preclinical research and clinical therapeutic strategies for CNS diseases.

## 2 Current epidemiology of Alzheimer’s disease

Attributing to population ageing and population growth, the global prevalence of AD is expected to reach 106.8 million cases (Brookmeyer et al., 2007). Moreover, driven by both increased life expectancy and declining fertility, the number of AD patients will continue to increase in the future (Lutz et al., 2008; Collaborators and G.B.D.D., 2019). According to the 2019 official death certificates, AD has become the fifth leading cause of death among people aged 65 years and older in the United States and the mortality has further deteriorated with the COVID-19 pandemic in early 2020 (Alzheimer’s Disease Facts and Figures, 2021). AD patients are in a state of dependence and disability for a long time before death, which has a serious impact on the public health of society. The global annual expenditure on health care for dementia is as high as 1 trillion dollars, of which AD accounts for the majority (Livingston et al., 2020). Therefore, AD has been identified by the World Health Organization as a global public health priority.

Up till the present moment, the etiology of AD remains poorly defined, although aging, genetics, family history and some modifiable risk factors still influence the incidence of AD. Research reports from the World Health Organization and the National Academy of Medical Sciences have pointed out that except for inherent risk factors such as age cannot be altered, other risk factors such as physical activity, blood pressure, diet, smoking and being social can be modified to reduce the risk of AD and cognitive decline (Blazer et al., 2015). Brain health is also significantly influenced by cardiovascular health, and many of the factors that aggravate the risk of cardiovascular disease are also associated with a higher risk of AD (Samieri et al., 2018). Some other studies have shown that midlife obesity (Kivimaki et al., 2018), high cholesterol (Solomon et al., 2009) and diabetes (Vagelatos and Eslick, 2013) all bring an increased risk of AD. Details of the latest risk factors affecting AD and interventions are summarized in Table 1. These non-pharmacological interventions may reduce behavioral symptoms and improve overall quality of life in some patients with mild AD.

**TABLE 1:** Latest Risk factors and preventive measures for Alzheimer’s disease.

Risk of Alzheimer’s disease		
Inherent risk factors	Modifiable risk factors	Prevention/Intervention
Age	Excessive alcohol consumption	Limit alcohol use
Genetics	Head injury	Protection of the head against injury
Family history	Air pollution	Reduce exposure to air pollution
	Less education	Provide formal education
	Hypertension	Vascular health
	Hearing impairment	Protection of ears/Encourage use of hearing aids
	Smoking	Quit smoking
	Obesity	Reduce obesity in midlife/Exercise
	Depression	Active treatment/Remain optimistic
	Physical inactivity	Physical activity
	Diabetes	Balanced diet
	Infrequent social contact	Increase social/cognitive
	Poor sleep quality	Improve sleep

An overview of risk factors and intervention which have been implicated in Alzheimer’s disease to date. These risk factors include three inherent risk factors and twelve modifiable risk factors. Actively addressing these risk factors might prevent and delay the occurrence and progression of AD.

## 3 An update on pathophysiology of Alzheimer’s disease

### 3.1 Aβ peptide and plaques

In the mid-1980s, Aβ protein was found to be the primary pathological component of amyloid plaques isolated from tissues in the brains of AD patients (Glenner and Wong, 1984; Masters et al., 1985). In a subsequent study, it was discovered that the amyloid precursor protein (APP) gene encoding type 1 transmembrane protein is located on chromosome 21, and the gene encoding Aβ protein is also located on the same chromosome (Goldgaber et al., 1987; Robakis et al., 1987). Since then, most of the research has focused on Aβ peptide and APP, and the amyloid cascade hypothesis has been derived. The amyloid cascade hypothesis, based on a combination of histopathological observations and combined genetics, has dominated research for the past 2 decades.

APP is a transmembrane protein that is highly expressed in the central nervous system (CNS) and has three domains, including a longer extracellular domain, a shorter intracellular domain, and a cell membrane domain. It has been shown that APP plays a significant role in neuronal synaptogenesis, dendritic sprouting, neuronal protein transport and cell adhesion, with an integral effect on neuronal function (Zheng and Koo, 2006; Muller et al., 2017). Aβ peptide is a small peptide composed of 38–43 amino acids and is produced in the sequential cleavage of the APP by β- and γ-secretase complexes (Haass, 2004). In normal physiological conditions of brain neurons, about 90% of APP is first cleaved in the middle part of the Aβ region by the α-secretase to generate a N-terminal secreted APP (sAPPα) and the carboxyl terminal fragments of 83 amino acids (CTF83 or CTFα) (Gallardo and Holtzman, 2019). sAPPα is a large, soluble secreted fragment of APP that is involved in neuronal plasticity and protects hippocampal neurons from excitotoxicity and Aβ toxicity (Furukawa et al., 1996). The CTF83 is further cleaved by γ-secretase to generate a small P3 peptide and APP intracellular domain (AICD) (Gu et al., 2001). Notably, P3 is a truncated Aβ peptide that evolves into a β-hairpin conformation after cleavage by γ-secretase and then assembles into a fibrillar aggregates that is unstable in the smaller oligomer, so that it would be devoid of the neurotoxic effect of Aβ (Dulin et al., 2008). ACID, released from the membrane along with P3, was not described until 2000 (Passer et al., 2000) and might play a role in nuclear signaling transduction (von Rotz et al., 2004). There has been evidence that free AICD can regulate the transcription process of multiple potential target genes, including APP itself, KAI1, neprilysin, P53, LRP1, BACE1, and EGFR (von Rotz et al., 2004; Baek et al., 2002; Pardossi-Piquard et al., 2005; Zhang et al., 2007; Liu et al., 2007). ACID may be a cell death mediator via increasing the expression of P53, which lowers the cellular threshold of apoptosis, thus increasing the resistance of neuronal cells to toxic and apoptotic stimuli (Passer et al., 2000; Giliberto et al., 2008). Furthermore, Matrone et al. knocked in the dysfunctional motif Y682G in the AICD of APP transgenic mice and found that the mice exhibited deficits in cognitive function and muscle strength (Matrone et al., 2012), which may be due to the critical cholinergic function of intracellular ACID. Collectively, this processing pathway initiated by α-secretase cleavage is known as the non-amyloidogenic processing pathway.

**FIGURE 1:**
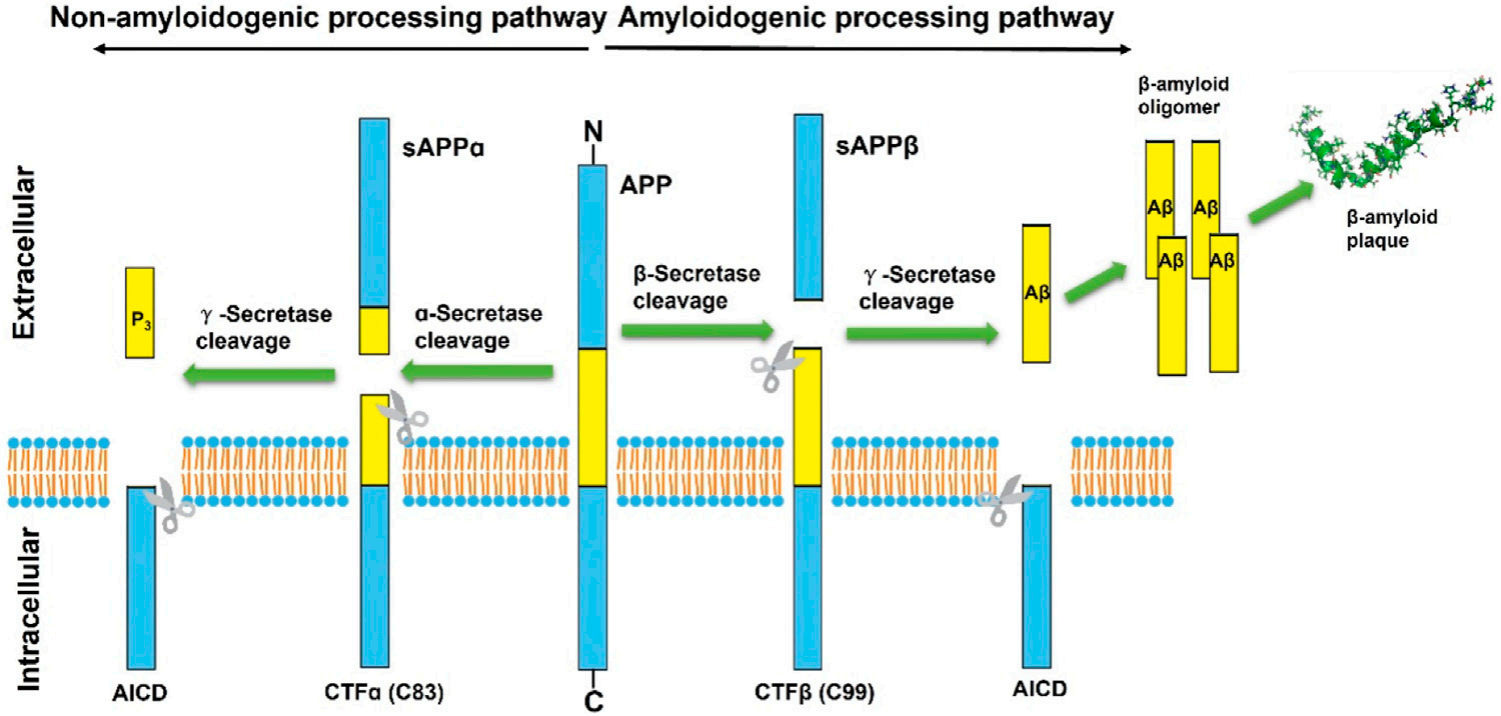
Schematic diagram of classical proteolytic processing of amyloid precursor protein (APP) within the non-amyloidogenic and amyloidogenic pathways. The APP is initially cleaved by α and β-secretases, respectively. In the classic amyloidogenic processing pathway, APP is continuously cleaved by β-secretase and γ-secretase, resulting in insoluble Aβ deposition. In contrast, APP cleaved by α-secretases produces a truncated Aβ peptide, which does not aggregate and has no neurotoxicity.

Correspondingly, the amyloidogenic processing pathway in which APP is continuously cleaved by β-secretase and γ-secretase to generate insoluble deposits of Aβ (Figure 1). β-secretase, also known as Beta-site amyloid precursor protein cleaving enzyme-1 (BACE1), is a membrane-anchored aspartyl protease of the atypical pepsin family, which was identified by Vassar et al., in 1999 (Sinha et al., 1999; Vassar et al., 1999). BACE1 is a type I transmembrane protein that forms a subfamily of membrane-anchored aspartyl proteases together with Beta-site amyloid precursor protein cleaving enzyme-2 (BACE2) (Yan, 2017), a membrane-bound secretase with 75% homology and the same domains (Sun et al., 2005). The difference is that BACE1 is widely expressed in neurons, oligodendrocytes, and astrocytes of the brain, while BACE2 is thought to be mostly existed in peripheral tissues and expressed extremely low in the brain (Zhao et al., 2007; Esterhazy et al., 2011). A follow-up study by Zetterberg et al. (2008) found that BACE1 activity was elevated in mild cognitive impairment (MCI) subjects and in AD patients, and that there was a positive correlation between BACE1 activity in cerebrospinal fluid (CSF) and total tau levels.

During initiation of Aβ peptide production, β-secretase cleavage releases most of the ectodomain structure of APP into soluble sAPPβ and generates membrane-bound CTFβ (C99). Although sAPPβ only lack the Aβ1-16 region in structure compared with the aforementioned sAPPα, their effects are essentially different. SAPPβ may bind to Death Receptor 6 (DR6), trigger activation of caspase-3/6, and mediate neuronal cell death and axonal branch pruning (Nikolaev et al., 2009). CTFβ is further cleaved by γ-secretase at several potential transmembrane positions, resulting in Aβ peptides of 38–43 amino acids in length. Aβ exists and accumulates in different forms and eventually aggregates extracellularly to form fibrils and plaques. Among them, Aβ42 is more fibrotic and insoluble than Aβ40, so Aβ42 is more common in deposited plaques. Although Aβ can be generated in several subcellular compartments within the cell, the majority of Aβ is secreted out of the cell (Zhang et al., 2011). Extracellular aggregation of Aβ forms diffuse Aβ plaques and the dense-core plaques, which are particularly present in the hippocampus region. The deposition of dense-core plaques is commonly surrounded by microglia and reactive astrocytes, which in turn aggravate neurotoxicity and synaptic loss (Gallardo and Holtzman, 2019). The dense-core plaques also referred to as neuritic plaques, which are distortions in neuronal axons associate with dystrophic neurites. And the extracellular deposits of fibrillar Aβ could form senile plaques and accelerate the hyperphosphorylation of intracellular tau protein, which also induces neurotoxicity (Zhang et al., 2021). This has become one of the classic hallmarks of the histopathology of AD and has been replicated and studied in multiple AD mouse models. Therefore, many AD drugs targeting Aβ production or eliminating amyloid plaques have been developed, but none of them have been approved so far (Hung and Fu, 2017).

### 3.2 Tau protein and neurofibrillary tangles

While some studies demonstrated that Aβ abnormality precedes other AD-related pathologies, and that Aβ plaque deposition is necessary for AD progression, it is not sufficient to cause significant neuronal and synaptic damage, implying that amyloid cascade hypothesis is insufficient to explain many aspects of AD pathogenesis. A growing body of research is showing that the main component of neurofibrillary tangles (NFTs), another classic pathological feature of AD, is the hyperphosphorylated tau protein (pTau), which has sparked a rush to shift research targets from Aβ to tau (Giacobini and Gold, 2013). Today, the study of pTau aggregation showed that NFT deposition is closely correlated with the dysfunction of synapses and neurons, which can easily lead to memory impairment in AD patients.

Tau, a microtubule-associated protein located on the surface of microtubules, expresses primarily in neurons, and involves in the regulation of neural microtubule stability (Kempf et al., 1996). Microtubules are paramount components of the cytoskeleton and pathways for transporting organelle, and tau proteins improves the stability of microtubules by enhancing the stacking structure of microtubules (Ashrafian et al., 2021). In accordance with neurophysiological studies, neurons have polarized structure of axons and somatodendritic compartment, and microtubule-associated proteins play a major role in the polarity characteristics of neurons, such as tau protein localized to the axons *in situ*, and MAP-2 localized to cell bodies and dendrites (De Camilli et al., 1984; Binder et al., 1985). It is because of the special affinity of tau protein for microtubules that pTau inhibits the transport of kinesin on axons and thus triggers AD symptoms (Brady and Sperry, 1995). From the physiological mechanism, the phosphorylation of tau is essential for the normal physiological function of neurons. Ksiezak-Reding et al. (1992) found that normal brains contain 2–3 mol of phosphates per molecule of tau protein; while in the autopsied AD brains, tau protein contains 6–8 mol of phosphates per molecule, with much higher levels of phosphorylation than normal brains. This phenomenon indicated that numerous sites of normal tau have undergone post-translational modification, i.e., hyperphosphorylation during the development of AD (Matsuo et al., 1994). Among them, the altered phosphorylation of serine and threonine residues at sites cause tau to be released from microtubules to form tau dimers, which then form tau oligomers and aggregate to form paired helical filaments (PHF) structures, ultimately leading to the formation of large numbers of NFTs in the brains of AD patients (Grundke-Iqbal et al., 1986). Previous reports have also suggested that tau oligomers are more likely to cause neurotoxicity than tau aggregates and play a more prominent role in the propagation of neural networks (Chong et al., 2018). The abnormal conformation accumulates in the somatic dendritic region, then first appears in the entorhinal cortex, and propagates across synapses in a time-dependent manner through anatomically connected neuronal circuitry, eventually reaching the hippocampus and other brain regions (Liu et al., 2012). The standpoint is also confirmed in animal models by the injection of tau oligomers in the mouse hippocampus that the transmission of tau pathology from one region to another. Lasagna-Reeves et al. (2012) injected tau oligomers purified from AD cerebral cortex into the hippocampus of wild-type C57BL/6 mice and found that positive staining of tau deposits was not restricted to the injection area but spread to areas such as cortex, corpus callosum and hypothalamus, resulting in inhibition of hippocampal long-term potentiation (LTP) and memory impairment.

In the course of AD progression, some scholars believed that an imbalance between the two enzymes is the root cause of pTau in brain of AD patients, because normal tau phosphorylation is regulated by the activity of protein kinase and protein phosphatase (Wang et al., 2007). Previously studies have also claimed that both glycogen synthase kinase 3β (GSK3β) and cyclin-dependent kinase 5 (CDK5) are able to cause abnormal tau phosphorylation occurred in different sites (Chong et al., 2018). Probably due to the overproduction of Aβ in the microenvironment, tau protein is prone to hyperphosphorylation and further oligomerization when it contacts with the increased activity of protein kinase. The differentially pTau have been caused different kinases at different sites, which have various effects on neuronal function. A study by Chun and Johnson (2007) supports that caspase-cleaved tau tends to aggregate to form fibrillary structure *in vitro* and *in situ*, when phosphorylated by GSK3β. Other than that, protein kinase A/C, caspase3, ERK2, and serine/threonine kinase have critical roles in the regulation of tau phosphorylation (Tiwari et al., 2019). Similarly, *in vitro* studies have found that tau is easily dephosphorylated by protein phosphatases such as PP1, PP2A and PP2B, but insufficient dephosphorylation of these protein phosphatases seems to exacerbate AD pathology in mammalian brain (Stojakovic et al., 2021).

### 3.3 Microglial activation and neuroinflammation

Obviously, amyloid plaque deposition and neurofibrillary tangles caused by hyperphosphorylated tau are crucial in the pathogenesis of AD, but the relationship between the two mechanisms is unclear, and there is no clear evidence as to whether Aβ plaque is upstream of tau pathology. There is plentiful evidence that the autoimmune system plays a key role in the AD pathogenesis, possibly explaining this association (Lane et al., 2018). The elevated levels of inflammatory factors in brain with AD patients and identification of AD risk genes associated with innate immune function suggest that neuroinflammation consisting of the activation of both microglia and astrocytes may play an indispensable role in the pathogenesis of AD (Leng and Edison, 2021). Microglia, as the main immune effector cell of neuroinflammation, has become one of the main research targets for scientists.

#### 3.3.1 Genetic association of microglia with AD risk genes

In recent years, genome-wide expression profiling data and large-scale epigenome-wide association studies (EWAS) combined with a large number of functional studies have revealed that microglia are different from tissue macrophages or other immune cells, and have unique nervous system regulatory capabilities, including neurotrophic support and synaptic regulation (Wes et al., 2016). Notably, microglia are widely distributed in the neural tissue of the brain and form an anatomically reticular network capable of rapidly responding to modifications in the local microenvironment via ATP mediation (Davalos et al., 2005). On this basis, we speculate that the microenvironment in the brain and the functional activity of most neurons may be regulated by microglia. Mounting evidence verified that microglia maintain homeostasis and self-renewal under normal physiological conditions, protecting against Aβ accumulation and facilitating the clearance of these deposits around neuron (Lee and Landreth, 2010; Arranz and De Strooper, 2019). Genetic data from human AD also further confirmed that most of the risk genes associated with AD are highly expressed in microglia and that many are expressed selectively or preferentially (Cuyvers and Sleegers, 2016). For example, although apolipoprotein E (ApoE) affects the occurrence and progression of AD through various mechanisms, its momentous role is to stimulate the phagocytosis of Aβ polymers by microglia, shrank the load of Aβ plaques, and alleviate the inflammatory response (Terwel et al., 2011). Combining large-scale genome-wide survival analysis with endophenotypic association analysis, Huang et al. found that lower SPI1 expression may reduce the risk of AD by attenuating pro-inflammatory microglia; Specifically, SPI1 encodes a transcription factor PU.1 that is critical for microglial development, and the expression level of PU.1 regulates microglial functions, including proliferation, survival, and differentiation (Huang et al., 2017). The research also showed that the expression of colony-stimulating factor 1 receptor (CSF1R) was reduced after knock-down of SPI1. Actually, previous studies have shown that inhibition of the CSF1R at a lower level in 3xTg-AD mouse model can eliminate most of the number of microglia associated with β-amyloid plaques and significantly improve the cognitive ability of mice (Dagher et al., 2015). All these reveal the genetic association between AD risk genes and microglia expression genes, and its functional mechanism needs to be further dissected.

**FIGURE 2:**
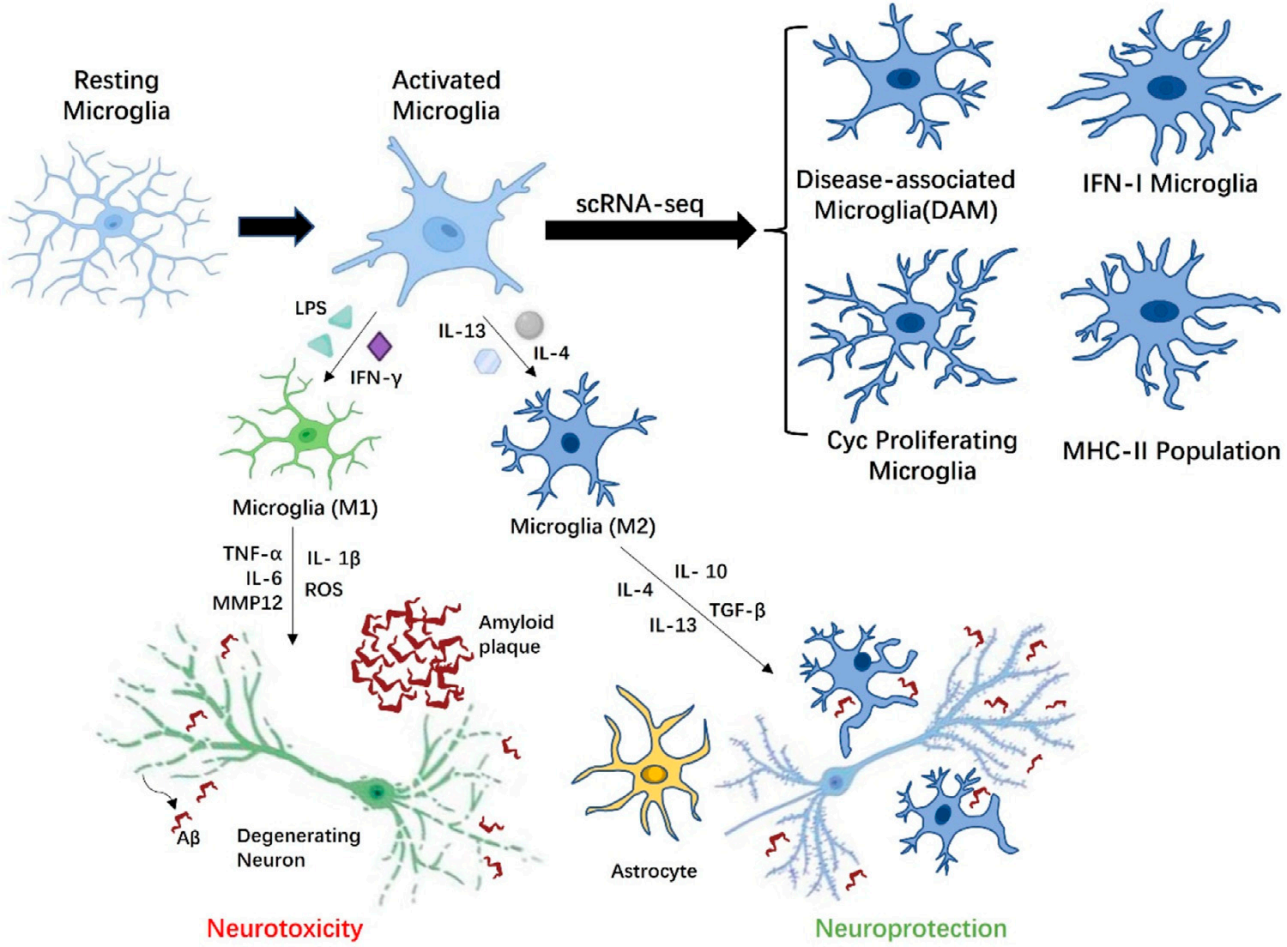
Microglia with different phenotypes participate in AD related pathological activities. Depending on the stimulation of AD microenvironment, resting microglia can be transformed into M1 phenotype microglia with neurotoxicity or M2 phenotype. microglia with neuroprotection. Under the influence of amyloid beta (Aβ) plaques, activated M1 microglia overproduce proinflammatory cytokines (IL-1β, IL-6, and TNF-α), which have toxic effects on neurons. M2 microglia are involved in modulating the release of anti-inflammatory mediators (TGF-β, IL-4, and IL-10), thereby reducing inflammation. Moreover, M2 microglia also cleared Aβ peptides by phagocytosis, limiting the damage of Aβ peptides to the adjacent neuropil to the greatest extent. Microglia could indirectly affect neuronal activity through astrocytes. Finally, scRNA-seq results indicated multiple activation states in microglia are context-dependent in response to Aβ accumulation, including disease-associated microglia (DAM) localized in the vicinity of the Aβ plaques, Cyc proliferating microglia, IFN-I microglia, and MHC-II population.

In the histopathological development of AD, researchers generally believed that the aggregates of Aβ peptides and tau, and neuronal death trigger aberrant activation of homeostatic microglia-reactive microglia, and the unbridled microglia activity may be extremely harmful to neurons. Although microglia may have a positive effect on Aβ phagocytosis, the long-term presence of Aβ causes microglia to abnormally activate and express a variety of inflammatory receptors. It is worth mentioning that Aβ-plaque-associated microglia are thought to play a principal role in promoting neuroinflammation in AD disease models (Butovsky and Weiner, 2018). EI Khoury et al. (2003) used macrophages and microglia from CD36-deficient mice to demonstrate that CD36 is an inflammatory mediator of microglia response to fAβ and can induce activation of these cells to produce ROS, TNFα, IL-1β and some chemokines. Genome-wide association studies (GWAS) of late onset AD have also proved that a rare Arginine-47-Histidine (R47H), a variant of the microglial-expressed innate immune receptor the triggering receptor expressed on myeloid cells 2 (TREM2), impairs the binding of TREM2 to phospholipid ligands, rendering microglia to be unable to respond to Aβ deposition through TREM2 and limiting neuronal degeneration (Wang et al., 2015). This weakening of the response barrier may promote the spread and invasion of Aβ amyloid in the early stages of AD, triggering neuroinflammation (Wang et al., 2016). Moreover, it is widely accepted that Aβ can also increase the concentration of extracellular ATP, which activates P2X7R to trigger the release of IL-1β and enhances excitatory synaptic activity (Sanz et al., 2009; Martin et al., 2019). Amusingly, this is not consistent with the latest finding that the lack of P2X7R reduced Aβ pathology by reducing chemokine (e.g., CCL3, CCL4 and CCL5) release in APP/PS1 mice. During neuroinflammation progression in AD, extracellular ATP induces P2X7R hyperactivation in microglia, which triggers the activation of microglia, decreased phagocytic activity and released various neurotoxic molecules, including TNF-a, ROS, MMP-9,IL- 1β and IL-6 etc. (Vasic et al., 2019). An increase in the release of these factors further induce and exacerbate a broad neuroinflammatory response and neuronal injury. Like macrophages, it is generally believed that the activation of microglia is divided into classical activation M1 and alternative activation or acquired deactivation M2. M1 microglia have a pro-inflammatory state, which can not only produce pro-inflammatory factors (e.g., TNF-α, IL-6, IL-1β) and chemokines (MCP-1), but also enhance the release of ROS and the excitotoxicity of glutamate, thereby resulting in neuronal and other damaging effect (Appel et al., 2011). Similarly, M1 microglia also express and produce matrix metallopeptidase 12 (MMP12) and a large amount of integrins, which eventually induce high levels of inflammation and neurotoxicity in brain. The other way round, M2 microglia are thought to have anti-inflammatory and healing properties, promoting the secretion of anti-inflammatory factors (e.g., IL-10 and TGF-β) and growth factors (e.g., IGF-1, FGF, and CSF1) as well as neurotrophic factors (e.g., NGF, BDNF, and GDNF) (Colonna and Butovsky, 2017). It is well known that neurotrophic factors not only have supportive and protective effects, but also regulate synaptic strength and plasticity, which are essential for the development and maintenance of nerves. While the activation status of M1 and M2 contributes to our understanding the activity of microglia *in vivo*, more and more researchers believe that the M1/M2 pattern is not sufficient to better describe the activation phenotype of microglia in AD (Figure 2).

#### 3.3.2 The activation state of microglia during AD progression

Recent advanced single-cell RNA sequencing (scRNA-seq) and single-nuclei RNA sequencing (snRNA-seq) analyses has also revealed from a transcriptome perspective that multiple activation states in microglia are context-dependent in response to Aβ accumulation, including disease-associated microglia (DAM) localized in the vicinity of the Aβ plaques, Cyc proliferating microglia, IFN-I microglia and MHC-II population (Chen and Colonna, 2021). The trajectory analysis in the study further suggests that microglia eventually form four independent trajectories in a progressive manner from homeostasis. Among them, activated microglia with DAM-like characteristics not only play a critical role in CNS development, but may also have efficient phagocytosis and clearance of pathological aggregates in AD brain. In contrast, homeostatic microglia were observed to activate and generate two-stage DAM in a TREM2 independent and TREM2 dependent manner in the CK-p25 model of neurodegeneration (Mathys et al., 2017). There are significant differences in gene expression characteristic of the different stages of DAM produced by the two modalities, so it remains to be determined whether the role of DAM in the microenvironment is a double-edged sword. Studies have also indicated that microglia with DAM-like characteristics are affected by the deletion of TREM2 and ApoE. Trem2 is involved in the proliferation and survival of microglia, and the deficiency of Trem2 results in a decrease in the number of microglia surrounding Aβ plaques and causes additional neuroinflammation (Wang et al., 2015). ApoE is the main apolipoprotein lipid in CNS, which is produced by microglia under pathological conditions. The absence of ApoE could reduce the toxicity of Aβ, thus exerting a neuroprotective effect and affecting the metabolism of microglia (Chen et al., 2021). IFN-I and MHC-II microglia were identified in the hippocampus of the CK-p25 mouse model of severe neurodegeneration as distinct subsets from DAM (Mathys et al., 2017). The temporal tracking approach in the research also revealed a transition of microglia from a state of marked proliferation in early response to a state of enhanced immune response in late response, and there is a significant correlation in gene expression between the two microglia. For Cyc proliferating microglia, Wang et al. found a proliferative microglia cluster expressing Ki67 around Aβ plaque in 5XFAD mice in 2016 (Wang et al., 2016). The scRNA-seq research further confirmed that the Cyc proliferating microglia cluster is mainly in the G2/M phase and expresses associated markers such as Mki67, Mcm5, Top2a, Cenpe, Tpx2 etc. (Wang et al., 2020). The groundbreaking finding reflects changes from “resting” or homeostatic microglia engaged in surveillance activities in the normal neural microenvironment to a disease state stage in the AD microenvironment, in which Trem2 may play an essential induction role. This greatly deepens our understanding of the activation state of microglia during AD progression.

In the microenvironment of the AD brain, both resting and activated microglia are highly dynamic (Colonna and Butovsky, 2017). Local tissue damage or inflammatory stimuli induce changes in the shape and movement of microglia and trigger a series of functional roles of microglia. There is considerable experimental evidence that microglia are involved in amyloid plaque clearance, neurofibrillary tangles, and synaptic loss through activation of complement in human AD brain tissue (Zanjani et al., 2005; Stephan et al., 2013). Microglia are the major source of plaque associated C1q, and in a mouse model of β-amyloidosis, elevated levels of C1q expression triggers activation of the classical complement cascade by binding to Aβ (Jiang et al., 1994; Hong et al., 2016). C1q mediates early synaptic loss even before significant plaque deposition increases, and activation of the classical complement cascade in conjunction with Aβ drives synapse clearance by microglia through CR3. This means that although microglia and complement activation are considered to be mainly involved in protecting the neuropathology around the plaque, after being affected by Aβ peptide with synaptic toxicity, microglia will become a cellular mediator of synaptic loss, subsequently neuroinflammation and cognitive decline. Additionally, knockdown or inhibition of complement-activated central complement component C3 or downstream complement activation fragments may also provide the clearance of plaque and neuroprotection, as well as protect against cognitive decline (Hong et al., 2016; Shi et al., 2017). Nevertheless, the role of C3 in AD remains controversial. Maier et al. (2008) developed a complement C3-deficient amyloid precursor protein (APP) transgenic AD mouse model (APP; C3−/−) and found that C3 deletion resulted in increased Aβ deposition in 17-month-old mice and decreased NeuN-positive neurons in the hippocampus, demonstrating that complement C3 has a beneficial neuroprotective role. As such, the consequences of complement C3 in amyloid plaque, related synapses, and neurons in the aging AD brain, remains to be further explored. On the other hand, although the mechanism remains poorly understood, inhibition of complement activation may also be beneficial to alleviate the pathological manifestations of tau protein (Colonna and Butovsky, 2017). Britschgi et al. (2012) indicated that a soluble complement receptor-related gene y (sCrry), a natural inhibitor of C3, can reduce the phosphorylated tau levels of AT8 epitope in the brain of P301L mutant tau/sCrry double transgenic mice. Significantly, in the absence of exogenous trigger of inflammation, the authors also observed a strong link between microglia activation and the number of AT8-positive cells in P301L tau transgenic mice. Besides, experiments using a rapid tau propagation mouse model showed that microglia promoted the spatial propagation of tau protein in the functional area of the brain through phagocytosis and exocytosis (Asai et al., 2015). Specifically, microglia phagocytize the diseased neurons or synapses containing tau protein, and then secrete tau protein in the form of exosomes, so as to realize the effective propagation of tau protein among neurons. Strikingly, Asai et al. also indicated that the expression of pro-inflammatory and anti-inflammatory cytokines and the secretion of tau by microglia were significantly decreased in the brain by pharmacological depletion of microglia (PLX3397, an inhibitor of CSF1R) or inhibition of exosome synthesis [neutral sphingomyelinase 2, nSMase2 (Trajkovic et al., 2008)].

Taken together, microglia in the brain act as a double-edged sword, both facilitating pathogen clearance and potentially triggering neurotoxic factors by activating inflammatory mediators. These findings strengthen the close relationship between abnormal activation or dysfunction of microglia and the partial neuroinflammation it triggers and the pathogenesis of AD, suggesting that the harmful effect of activated microglia may be a universal feature, which may be a contributing factor rather than a concomitant manifestation.

### 3.4 The dysfunction and loss of synapses

In the complex neuronal network in the brain, the output and input of neural electrical or chemical signals are determined by the combined activity of each neuron and synapse. The change of individual neuron or synapse will affect its signal transmission efficiency and the plasticity of local synapses, thus influencing the normal function of other neurons connected to it. Synaptic plasticity is also considered to underlie the anatomical structure of learning and memory in the healthy brain (Spires-Jones and Hyman, 2014). This theory was initially supported by the discovery of LTP in the hippocampal dentate area of the anaesthetized rabbit (Bliss and Lomo, 1973). This concept was further emphasized by the hypothesis of synaptic plasticity and memory formation by Takeuchi et al. (2014). Notably, LTP and long-term depression (LTD) of hippocampal synaptic transmission remain the prime candidates for probing the synaptic plasticity during learning and memory in animals (Malenka and Bear, 2004). Given the neuroscientific rationale that neuronal and synaptic function is the basis of cognitive performance, it is widely recognized that the damage or loss of neurons and synapses is the best correlate of cognitive decline in AD (Blennow et al., 1996; Spires-Jones and Hyman, 2014). While the specific molecular mechanisms causing synaptic degeneration in AD have not been fully explained so far, immunohistochemical and electron microscopic studies showed that the neuronal and synaptic loss also were the two main pathological causes of cerebral cortical atrophy (Serrano-Pozo et al., 2011), so alterations in normal synaptic function may remain central to the disease progression.

#### 3.4.1 Aβ, tau protein and the dysfunction of synapses

In 2011, Serrano-Pozo et al. classified the neuropathological hallmarks of AD as “positive” lesions including the deposition of amyloid plaques, neurofibrillary tangles containing hyperphosphorylated tau, microglial abnormal activation with astrogliosis, and neuroinflammation, and “negative” lesions comprising the neuronal and synaptic damage or loss. Though each lesion is a characteristic pattern of AD, the mechanistic associations between these “positive” lesions and changes in synaptic plasticity remains unclear. Colom-Cadena et al. (2020) thought that synaptic loss is a downstream effect of “positive” lesions during AD progression. Synaptic loss exceeds existing neuronal loss within a particular cortical area which also suggests that synaptic loss is not just caused by neuronal loss, but even predates neuronal loss (Scheff et al., 1990; Ingelsson et al., 2004). The first concern is, of course, the senile plaques formed by Aβ accumulation, one of the classic pathological lesions of AD, which can lead to synaptic plasticity deficits, abnormal patterns of neuronal activity and memory dysfunction (Ashe and Zahs, 2010). Although under normal microenvironment, physiological levels of Aβ will promote neuronal activity through presynaptic potentiation in the form of positive feedback loops, a small chronic increase in Aβ will eventually lead to synaptic depression (Abramov et al., 2009), let alone pathological elevates. Pathologically elevated Aβ severely disrupts signaling activity in circuitry and neuronal networks. The hAPP_FAD_ mice model also confirms that hAPP/Aβ triggers aberrant excitatory neuronal circuit activity and compensatory inhibitory responses in hippocampal circuits, and both alterations lead to complex imbalances in wide neuronal circuits activity and cognitive decline in the brain (Palop et al., 2007). Of interest, cognitive decline or synaptic loss in AD patients was not most strongly associated with plaque load. Perez-Nievas et al. (2013) found that a substantial proportion of elderly individuals without cognitive impairment had abundant amyloid-β plaques at autopsy, and phase I clinical trial of immunisation to remove plaque also showed that reducing plaque burden is not enough to halt cognitive decline in AD patients (Holmes et al., 2008). This seems to indicate a clear dissociation between AD pathological manifestations and cognitive status in some populations, and the accumulation of plaques in the brain does not appear to be associated with cognitive decline. Will the amyloid hypothesis be subverted? The answer is currently no. As has been proposed, soluble forms of Aβ are toxic to synapses, neuron, and cognition than the plaques themselves (Perez-Nievas et al., 2013; Spires-Jones and Hyman, 2014). In 1998, Lambert et al. reported fibril-free forms of small diffusible Aβ oligomers rapidly inhibits LTP in hippocampal neurons, which occurred without a lag (Lambert et al., 1998; Koffie et al., 2009). This affects spontaneous neurotransmitter release and synaptic plasticity as well as the alterations of synaptic excitatory transmission. Examination of excitatory synapse density in the APP/PS1 mice model using array tomography also found that oligomeric Aβ surrounding the senile plaques may interact directly at the synapses, resulting in significantly reduced postsynaptic densities (PSDs) and synaptic dysfunctione. This technological advance reconciles the competitive relationship between plaque and oligomeric Aβ as the synaptotoxic substances, specifically, plaque acts as a local reservoir for oligomeric Aβ, which is directly synaptic toxic. Extensive research also supports the above point of view that soluble oligomeric Aβ is the most toxic species affecting synaptic dysfunction in early AD (Cleary et al., 2005). The results in hippocampal slices of mice also support oligomeric Aβ induced synaptic loss and dysfunction and found that C1q acts as a key mediator to mediate synaptic elimination (Hong et al., 2016). Collectively, these changes can affect the intersynaptic mechanisms of neuronal conduction and disrupt the normal activity of synapses, thereby impairing the neural network activity throughout the brain.

In addition to soluble amyloid-β oligomers, hyperphosphorylated and misfolded tau may also play a decisive role in neuronal disruption and cognitive decline. Aberration conformation of tau causes both damage of normal function and gain of fibrillogenicity that results in aggregation, which are associated with synapse and neuronal loss. Hyman believed that synaptic changes induced by Aβ may be an vital contributor to early AD, while the pathological property of tau contributes more to synaptic degeneration and cognitive impairment in middle and late AD (Hyman, 2011). Several studies have also confirmed the idea that tau may play more critical roles in synaptic dysfunction compared to Aβ. Since normal tau proteins are usually localized to both presynaptic and postsynaptic terminals, pathological accumulation of aberrant tau occurs on both sides of the synapse during AD progression, directly affecting synaptic function (Tai et al., 2012). This appears to be similar to the observation that oligomeric Aβ accumulates at the synapses, where tau forms oligomers and accumulates after being hyperphosphorylated and ubiquitinated under pathological conditions (Koffie et al., 2012; Tai et al., 2012). There is increasing evidence also suggests that p-tau oligomers tightly bound to PSD and cytoskeleton mediate synaptotoxicity in AD at least in part (Spires-Jones et al., 2009). More importantly, hyperphosphorylated tau reduces its affinity for microtubules, leading to destabilization of the microtubules and the increase of unbound tau, ultimately giving rise to neurofibrillary tangles (NFTs) and neuropil threads (NTs) (Stoothoff and Johnson, 2005). As AD progresses, NFTs formation and accumulation appear to follow the olfactory and limbic pathways of the allocortex, including the entorhinal cortex, the CA1 of the hippocampus, the amygdala, and its radiation regional (basal large cell complex) (Krstic et al., 2013). The synaptotoxic effects and NFTs caused by these abnormal post-translational modifications of tau might lead to cognitive deficits, which would then be further exacerbated as the affected neuronal network expands. When large areas of damaged neuronal cells die, the brain will face with some changes in brain parenchyma such as whole brain atrophy (Hung et al., 2016) and severe cognitive impairment.

#### 3.4.2 Microglia and the dysfunction of synapses

Microglia, previously thought to be modulator of neurodegeneration, have recently been shown to function beyond in neuroinflammation and release of neurotoxic molecules, but also in the phagocytosis of synaptic structures. Indeed, microglia play a critical role in the refinement of neural circuits, as important participants in pruning synapses during early brain development (Paolicelli et al., 2011). When in the pathological environment of AD, the proliferation of microglia and astrocytes in the brain is positively correlated with NFT burden, but not with amyloid plaque burden (Serrano-Pozo et al., 2011). Among them, microglia perform synapses elimination against dysfunctional neurons or neurons with partial fibrillary tangles. Hong et al. (2016) also found in their study that microglia in the CNS phagocytize synapses through complement-dependent mechanisms when challenged by synaptotoxic oAβ, meaning that microglia are potential cellular mediators of synaptic loss. Otherwise, their studies also directly challenge the idea that microglia are secondary events associated with neuroinflammation, that microglia and complement pathways may be involved in synaptic loss and dysfunction prior to plaque formation in early AD. Roy et al. (2020) using means of a highly specific blocking antibody method, also showed synaptic loss due to IFN stimulation of the complement cascade to drive activation of microglia in AD models in the absence of amyloid plaque pathology. These findings stand by the fact that amyloid load and tangles in human brains correlate poorly with cognitive severity or duration (Terry et al., 1991; Serrano-Pozo et al., 2011), suggesting that microglial activation and neuroinflammation are pivotal elements in the process of synaptic damage.

Recently, research by Lee et al. (2022) have subverted the traditional hypothesis that extracellular amyloid plaques are first formed and led to nerve cell death. The results indicated that the development of AD begins with the disorder of autolysosome acidification in nerve cells, which leads to the accumulation of β-amyloid protein and its precursor protein and the release of hydrolases into the cytoplasmic catabolic digested cells, eventually triggering the formation of extracellular amyloid plaques. This breakthrough finding may lead to a rethinking of the pathogenesis of AD and changes in subsequent treatment strategies. Hence, it is essential to dissect these interactions between the complex pathology and their concomitant concurrent signaling events for understanding synaptic loss and its relationship with cognitive decline.

## 4 DPSCs therapeutic potential in Alzheimer’s disease

Till date, only four medications including three acetylcholinesterase (AChE) inhibitors (donepezil, rivastigmine and galantamine) and N-methyl-d-aspartate (NMDA) receptor antagonist (memantine) are approved by the United States FDA for the treatment of cognitive impairment and dysfunction in the progression of AD. Despite decades of intense efforts by the pharmaceutical companies, all these synthetic drugs could only provide provisional symptomatic remission, and do not significantly improve disease progression. Just as the FDA accelerated approved in June 2021, Biogen’s monoclonal antibody, aducanumab, is used to alleviate amyloid plaque build-up in the AD brain, but there is not enough evidence that it can significantly reduce cognitive symptoms (SalwaKumar, 2021). As scientists continue to explore the pathogenic and pathophysiological mechanisms of AD and find that existing drug therapeutic interventions cannot cope with such complex pathological interactions (Traynor, 2015), there is a desperate need for future therapies to halt or reverse AD lesions. For this reason, given the limited regenerative capacity of the CNS and the current situation of irreversible nerve injury, transplantation of exogenous stem cells to replace the dysfunctional neural cells in the damaged CNS are coming up as most promising and potential alternative. The revolutionary discovery in recent years that stem cells could differentiate toward specific lineages, stimulate *in situ* neurogenesis, and secrete a variety of regulatory factors, implicates that they may replenish neurons destroyed in AD progression and regulate the affected microenvironment. Likewise, the advent and boom of stem cells tracer and 3D bioprinting techniques have ushered a new era in the stem cells treatment of AD.

In general, there are three types of stem cells commonly used for AD research, including embryonic stem cells (ESCs), induced pluripotent stem cells (iPSCs) and MSCs. However, the low availability and high variability of partial stem cells, along with ethical issues, may lead to uncontrollable transplant outcomes. What is worth mentioning, MSCs, a multipotent stem cells with a variety of sources, are capable of differentiating into non-mesenchymal lineages (Chakari-Khiavi et al., 2019). There are several sources of MSCs currently known, including bone marrow, adipose tissue, Wharton’s jelly, umbilical cord blood, and dental pulp. Intriguingly, most of the stem cells clinical trials registered with ClinicalTrials.gov for the treatment of AD also use MSCs (Table2). Here, we discuss the DPSCs, a more readily available MSCs population, that does not require invasive routine collection procedures of human organs or tissues and poses limited ethical concerns.

**TABLE 2:** Stem cells clinical trials for the discussed Alzheimer disease that are registered with ClinicalTrials.gov and signified as “not yet recruiting”, “recruiting”, “enrolling by invitation”, “active not recruiting”, “completed”, or “unknown status” (as of June 2022).

Row	Status	Study title	Stem cell type of intervention	Phase	Country
1	Recruiting	Allogeneic human mesenchymal stem cells for Alzheimer’s disease	Human mesenchymal stem cells	2	United States
2	Active not recruiting	Alzheimer’s disease stem cells multiple infusions	Allogeneic hMSC	1	United States
3	Unknown	Safety and efficiency of umbilical cord-derived mesenchymal stem cells (UC-MSC) in patients with Alzheimer’s disease	Human umbilical cord derived MSC	1/2	China
4	Completed	Safety and exploratory efficacy study of NEUROSTEM^®^ versus placebo in patients with Alzheimer’s disease	Human umbilical cord blood derived mesenchymal stem cells	1/2	Korea
5	Completed	The safety and the efficacy evaluation of NEUROSTEM^®^-AD in patients with Alzheimer’s disease	Human umbilical cord blood derived-mesenchymal stem cells	1	Korea
6	Completed	Lomecel-B infusion versus placebo in patients with Alzheimer’s disease	Longeveron mesenchymal stem cells	1	United States
7	Recruiting	The safety and the efficacy evaluation of allogenic adipose MSC-exos in patients with Alzheimer’s disease	Allogenic adipose MSC-exos	1/2	China
8	Not yet recruiting	Study to evaluate the safety and efficacy of AstroStem in treatment of Alzheimer’s disease	AstroStem	2	United States
9	Completed	A study to evaluate the safety and efficacy of AstroStem in treatment of Alzheimer’s disease	AstroStem	1/2	United States
10	Not yet recruiting	Exploratory efficacy study of NEUROSTEM^®^ in subjects who control group of NEUROSTEM^®^	Human umbilical cord blood derived mesenchymal stem cells	Not applicable	Korea
11	Recruiting	Follow-up study of safety and efficacy in subjects Who completed NEUROSTEM^®^ phase-I/IIa clinical trial	Human umbilical cord blood derived mesenchymal stem cells	1/2	Korea
12	Recruiting	Alzheimer’s autism and cognitive impairment stem cell treatment study	Bone marrow stem cell (BMSC)	Not applicable	United States
13	Not yet recruiting	Autologous stem/stromal cells in neurological disorders and disease	Autologous adipose stem cells	Not applicable	United States

### 4.1 General properties and cryopreservation of DPSCs

Teeth are composed of enamel of outer minerals and dentine of inner minerals (Sharpe, 2016). The dental pulp is softly vascularized connective tissue wrapped in the mineralized structures within the inner layer of the tooth. In 2000, dental pulp stem cells (DPSCs) are a type of permanent tooth-associated MSCs firstly isolated from the perivascular niches of dental pulp tissue of third molars impacted teeth by Gronthos et al. (2000). Apart from this, several new dental stem cell populations have been obtained, including stem cells from the pulp tissue of infant exfoliated deciduous teeth (SHED) (Miura et al., 2003), stem cells from apical papilla of immature permanent teeth (SCAP) (Sonoyama et al., 2008), and stem cells from periodontal ligament (PDLSCs) (Seo et al., 2004). Although these stem cell subpopulations may share the same source pathway, their phenotypic and functional properties are not all equal. And, DPSCs are usually derived from freshly extracted teeth for orthodontic purposes and impacted third molars, which are considered as an easily retrieved organic clinical waste (Seo et al., 2005). Therefore, their clinical applicability is attributed to multiple aspects, including their convenience of isolation, few ethical hurdles, and low immunogenicity (Huang et al., 2009).

**FIGURE 3:**
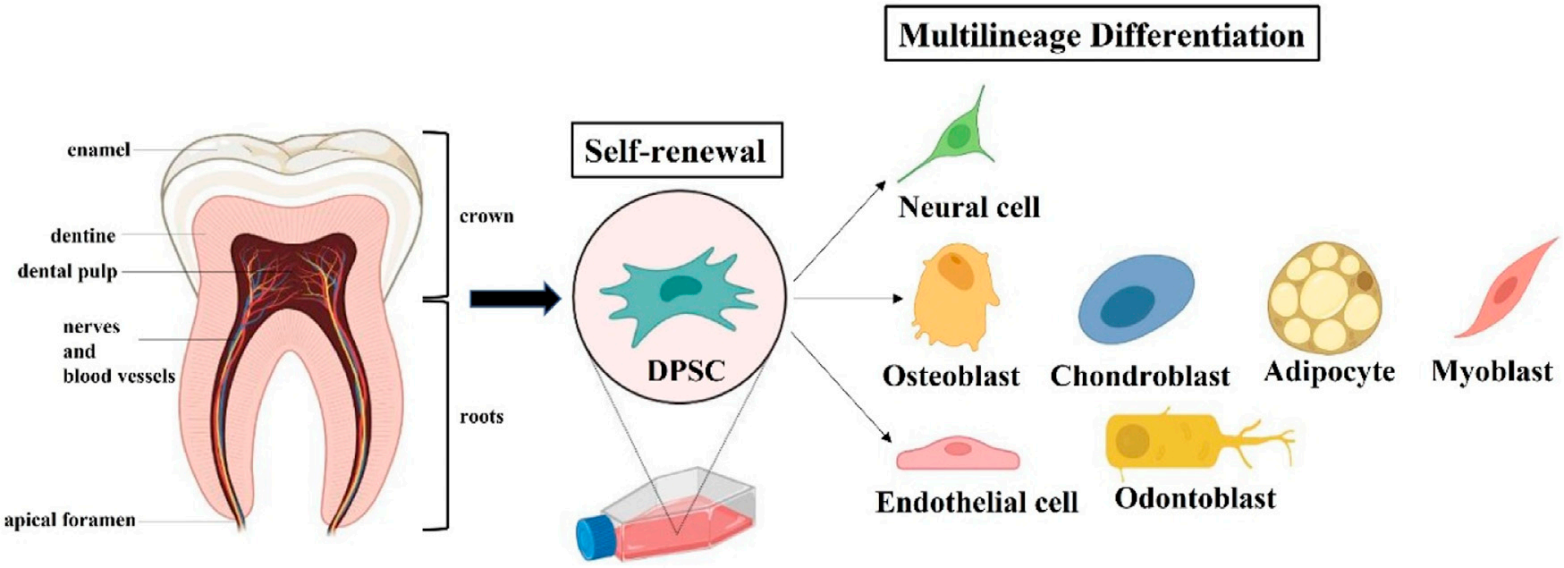
Extraction and Characteristics of dental pulp stem cells (DPSCs). DPSCs can be extracted from the tooth pulp tissue of adult molars and hold self-renewal and multilineage differentiation potential.

Under appropriate induction medium conditions, DPSCs have multilineage differentiation capacity and expression respective gene markers (Figure 3). But there is currently no unique biomarker expression profile to define DPSCs. Indeed, the immunophenotype of DPSCs *in vitro* has been reported that their expression of markers follows the criteria for defining multipotent MSCs stated by the International Society for Cell Therapy in 2006 (Gronthos et al., 2000; Dominici et al., 2006). Specifically, DPSCs express the common MSC-related markers CD73, CD90, CD105; but do not express the hematopoietic markers CD14, CD34, CD45, and HLA-DR. Under the standard cell culture conditions, DPSCs exhibit plastic adherence and spindle-shaped morphology, as do other types of MSCs. *In vitro* studies demonstrated that DPSCs presented significantly higher proliferation and differentiation in comparison with bone marrow mesenchymal stem cells (BMMSCs) and adipocyte stem cells (ASCs) (Huang et al., 2009; Tsutsui, 2020). DPSCs also exhibited the same expression pattern as ESCs-related markers OCT4, Nanog, SOX2 and MYC, which is rare to other types of MSCs (Liu et al., 2011). For these reasons, Brain organoids based on DPSCs are being developed to better explore the mechanism and treatment of AD via excellent *in vitro* models. It is worth noting that primary and induced immortalized DPSCs transplantation does not lead to tumor formation compared to the presence of potential tumorigenicity in ESCs and iPSCs (Wilson et al., 2015).

Of particular interest is the concept that cryopreservation allows the storage of whole teeth or DPSCs, which is an important consideration for the progressive realization of long-term clinical use. Studies have showed that, after uncontrolled or controlled cryopreservation, DPSCs could still maintain biological properties, including extraordinary proliferative and differentiative capabilities (Zhang et al., 2006; Kumar et al., 2015). In fact, long-term storage of stem cells has already been shown to be feasible. However, it is still the first time that the storage of intact teeth has reached the purpose of DPSCs banking. This is a more practical approach, which reduces the difficulty of manipulation before cryopreservation and the storage cost of the biobank, and not requires professional operators to immediately isolate DPSCs from various teeth (Perry et al., 2008). Recently, many countries have established good manufacturing process (GMP)-grade teeth bank for storing DPSCs just in case of need. Consequently, these excellent characteristics make DPSCs to be an exciting candidate for autologous stem cell transplantation therapies in AD.

### 4.2 Neural differentiation and replacement of DPSCs

As previously reported, AD is characterized by neuronal degeneration and consequent loss of function under the combined influence of complex pathological factors. Due to the negligible regenerative capacity of the CNS in adulthood, the introduction of exogenous stem cells to differentiate into neurons or other types of neural cells (microglia, astrocytes, and oligodendrocytes) in a specific microenvironment has become one of the promising strategies for the treatment of AD. In the past few decades, neural stem cells (NSCs), as a direct source of differentiation of nerve cells, has been extensively explored in the treatment of AD because of its great inherent advantages. Many preclinical animal models of AD have demonstrated positive results such as neurogenesis after NSC transplantation (Xuan et al., 2009). However, there are several critical issues that need to be addressed in the application of NSCs, such as the optimum cell source and long-term safety. NSCs are usually derived from human postmortem tissues or surgical specimens and are mainly distributed in the hippocampus and lateral ventricles of the brain, with low production and extremely difficult operating procedure (Palmer et al., 2001). The restricted accessibility of human NSCs have sparked an upsurge in the search for other suitable tissue-derived stem cells as plausible therapeutic strategies for AD.

Compared with other types of MSCs, DPSCs retain neuronal differentiation properties of the cranial neural crest cells from which they originate, so that they can differentiate toward functionally active neurons when exposed to appropriate neuronal inductive cues both *in vitro* and *in vivo*. As expected, on the one hand, DPSCs can express the pluripotent embryonic stemness-related markers like Oct-4, Nanog, and Sox-2. On the other hand, it can also spontaneously express early and mature neuronal-specific markers such as nestin, β-III tubulin, and neuronal nuclei (NeuN) at both the gene and protein levels (Kerkis et al., 2006; Luo et al., 2018b). More recently, there have been multiple *in vitro* neuronal differentiation protocols for DPSCs were developed. First of all, it is worth mentioning a 3D floating sphere culture system constructed by Pisciotta et al. (2018) to maintain the neural-related properties of hDPSCs. Their data demonstrated that hDPSCs and their progeny exhibited strong expression of β-III Tubulin and STRO-1^+^ after prolonged culture in the 3D floating sphere system compared to hDPSCs cultured in adherent conditions. Furthermore, hDPSCs cultured in spheroid also retain their immunomodulatory capacity, i.e., high expression of FasL. Previous studies have also shown that the expression of FasL by neurons in the CNS contributes to the protection of neural cells and the maintenance of relative immunosuppression (Moalem et al., 1999; Brunlid et al., 2007). Some of the more interesting studies have been the use of neurosphere formation to promote neurogenic maturation of DPSCs. Gervois et al. (2015) described for the first time the structural data of the microenvironment within neurospheres derived from DPSCs. Although there are still challenges in establishing fully functional neurosphere culture system of DPSCs, the results showed that differentiated DPSCs significantly have expression of neuronal and synaptic markers, improved communication network between adjacent cells, and partially differentiated into functional neurons. In addition to excellent *in vitro* neurodifferentiation performance, plenty of research using DPSCs transplanted into animal models have also demonstrated that DPSCs can differentiate into functional neurons or neurogliocytes in response to local microenvironmental cues *in vivo*. Through *in vivo* transplantation experiments, Arthur et al. (2008) demonstrated that DPSCs migrated and differentiated into neuronal derivatives and possibly integrated into neuronal networks within the developmental avian embryo model. Specifically, confocal image analysis showed that DPSCs had differentiated into neuron-like cells and exhibited the multiple neurite morphology, which co-localized with β-III tubulin. There are *in vitro* results also revealed that DPSCs highly expressed mature neuron marker NF-M in neural inductive media, while neuronal precursor nestin and early neuronal marker β-III tubulin were down-regulated. Electrophysiological findings also revealed voltage-gated Na^+^ channels expression of differentiated DPSCs. After that, the finding was further confirmed that these *in vitro* pre-differentiated hDPSCs can penetrate towards and integrate into the traumatically injured brain neural network structures by expressing neuron-specific markers and exhibiting voltage-dependent sodium and potassium channels, important for initiation of action potentials (Kiraly et al., 2011).

Other than that, given the better neuronal differentiation propensity of DPSCs in an appropriate microenvironment, exploring whether DPSCs can differentiate into spiral ganglion neurons (SGN) and dopaminergic cell-type are also pivotal for the clinical studies of DPSCs in other types of neurological disorders. After induction with BDNF, GDNF, NT-3 and SGN-inducing medium against DPSC and SHED isolated from non-carious deciduous and permanent teeth, it was found that these differentiated cells all expressed neuronal markers and SGN-specific markers, including Tuj1, GATA3 and NTRK2 (Gonmanee et al., 2018). The results of this *in vitro* study suggested that DPSCs have the potential based on stem cell therapy for neurosensory loss-related diseases. In 2014, Kanafi et al. (2014) demonstrated that DPSC showed differentiation towards functional dopaminergic neurons when cultured in midbrain cues mainly consisting of sonic hedgehog (SHH), fibroblast growth factor 8 (FGF8) and basic fibroblast growth factor (bFGF). The induced DPSCs not only showed enhanced expression of dopaminergic-neuronal markers like Nurr1, En1, and Pitx3, but also secreted dopamine constitutively. These experimental data revealed that DPSCs retain an excellent neuroplastic response to the neuronal microenvironment, making it a new, readily accessible, and useful source of neurogenesis *in vivo* that can be integrated into the CNS.

### 4.3 Neuroimmunomodulatory properties of DPSCs

Apart from providing neural substitution through differentiation directed by the environmental niche in brain, DPSCs also perform a significant role in immunomodulation at the site of tissue damage. DPSCs, similar to all MSCs, have been shown to exhibit exceptional immunomodulatory and immunosuppressive characteristics, especially downregulating the immune responses executed by T cells, dendritic cells (DC), NK cells and B cells (Chen et al., 2006; Yildirim et al., 2016). Furthermore, DPSCs are also able to prevent the inflammatory response by significantly shifting the quantities of the proinflammatory cytokines, as well as increasing the amounts of the anti-inflammatory cytokines (Yildirim et al., 2016). Many *in vitro* studies have confirmed the immunosuppressive activity of DPSCs, including their ability to affect T cell mediated reaction. Pierdomenico et al. have shown that DPSCs display an increased inhibition of T cell reactive activity when compared to BMMSCs (Pierdomenico et al., 2005; Gnanasegaran et al., 2017). Similar neuroimmunomodulatory effects were found in a study of DPSCs in the treatment of Parkinson’s disease (PD). As they have reported in this study, DPSCs significantly regulate secretion of anti-inflammatory cytokines (IL-10, IDO, and COX-2) against the surrounding inflammatory intensity when DPSCs had exposed in neuroinflammation microenvironmente. Upon introducing DPSC into the cell co-culture system, the researchers revealed that the secretory factors of DPSCs help reverse or neutralize the neuroinflammation initiated by both LPS and MPTP. The possible modulatory mechanisms involved include improvement of mitochondrial dysfunction and inhibition of toll-like receptor 4 (TLR4) and NLRP3 pathway activation. The existing studies have demonstrated that TLR4 and NLRP3 inflammasome affect the activation and clearance function of microglia in AD progression (Cui et al., 2020). Before that, Chang et al. (2005) also demonstrated that both LPS and TNF can activate the NF-KB signaling pathway in DPSCs, thereby triggering innate immune responses in DPSCs, which could participate in the regulation of inflammatory mediators, including TNF, IL-1, IL-6, and IL-8. Also, DPSCs have the ability to drive the polarization of macrophages toward M2 phenotype in response to tissue insult and inflammation, especially stimulation by IFN-γ (Zhang et al., 2009).

In a sense, any effect on the immune process *in vivo* involves the same mechanisms as *in vitro* activity. Therefore, these features of DPSCs *in vitro* are to be extrapolated in actual *in vivo* behavior, where they may regulate immune processes and inhibit inflammatory responses during tissue repair. For instance, it has been reported that conditioned medium from SHEDs (SHED-CM) can convert microglia from the proinflammatory M1 to the anti-inflammatory M2 phenotype in the brain of AD mouse (Mita et al., 2015). These effects include significant down-regulation of multiple pro-inflammatory cytokines (IL-1β and TNF-α), up-regulation of the expression of a major anti-inflammatory cytokine (IL-10), and suppression of Aβ1−40 induced oxidative stress. Meanwhile, treatment with SHED-CM inhibited glutamate-induced neuronal death and exerted multiple neurotrophic factors related to synaptic transmission and neuroprotection in AD, such as BDNF, IGF-1 and NGF. In another study, the intrahippocampal injection of DPSC-CM into mice with hippocampal damage significantly reduced neuroinflammation of the region and decreased the number of reactive astrocytes and microglia, whereas injecting BMMSCs-CM did not have anti-inflammatory effect (Venugopal et al., 2022). These comparative experimental results may be attributed to the superior immunomodulatory and anti-inflammatory efficacy of DPSCs. In addition, in other types of neurological diseases, DPSCs also show good immunomodulatory capacity. By transplanting the modified DPSCs into acute spinal cord injury (SCI), Albashari et al. found that it significantly prevented the activation microglia/macrophage and NF-κB signal pathway, and reduced the release of pro-inflammatory cytokines, alleviating tissue inflammation in the injured spinal cord and thereby promoting nerve repair (Albashari et al., 2020).

Up till the present moment, although the immunomodulatory mechanism of DPSCs may remain only incompletely elucidated, it is reasonable to believe that the personalized immunological priming and inflammatory control capabilities of neural crest derived DPSCs could open new ways for the application of stem cells in AD.

### 4.4 Neuroprotection and neurotrophy of DPSCs

When people study the MSC mechanism of action in neurodegenerative diseases, stem cell-based secreted factors are an inevitable key element. MSCs not only mediate its immunomodulatory and repair functions *in vivo* through direct cell-cell interactions, but also affect the overall microenvironment act in both autocrine and paracrine manners through secreted biological factors (Staff et al., 2019). In comparison to other MSCs, DPSCs significantly increase the expressions of BDNF, NGF, GDNF, NT-3, VEGF, and other neurotrophic factors, which could improve neuronal health and assist neurons to repair damage (Mead et al., 2013; Luo et al., 2018b). In 2015, studies by Tsiperson et al. (2015) have demonstrated that BDNF could regulate neuronal plasticity, proliferation, and survival *in vivo*, making it an essential trophic factor for early neuronal development and differentiation. Nerve growth factor (NGF) has been previously reported to support the survival and growth of neural cells and possess the ability to heal nerve injury (Aloe et al., 2015). These neurotrophin (NT) family growth factors are able to nourish and maintain neuronal plasticity and function in very minute concentrations in the nervous system of vertebrate (Huang and Reichardt, 2001). Hence, we are reasonable to hypothesize that the neuroprotective and neurotrophic effects exerted by engrafted DPSCs may be partly provided by these secreted cytokines.

Demicran et al. (2011) found that DPSCs not only regulate T cell-related immune responses, but also produce higher levels of hepatocyte growth factor (HGF) and transforming growth factor (TGF) via paracrine mechanisms, further supporting the above hypothetical view. More recently Jia et al. (Kumar et al., 2015) reported that HGF is a core functional factor secreted by hUC-MSC and plays an important role in MSC-mediated repair of damaged neurons in AD mice, at least in partially via activating cMet-AKT-GSK3β signaling pathway (Jia et al., 2020). It is further confirmed through the animal model experiments that intrahippocampal administration of DPSCs-CM upregulated the mRNA expressions of neuronal survival factors, including BDNF, GDNF, CNTF, VEGF and NGF (Venugopal et al., 2022). The authors believed that the neuroprotection, enhanced neurogenesis, and cognitive performance observed in the experimental group might at least partially attributed to the effects of neurotrophic factors secreted by DPSCs. As discussed in the study of Leong et al. (2012), functional recovery resulting from transplantation of DPSCs into the brain of poststroke animals may be modulated through DPSC-dependent paracrine effects. In a coculture model, DPSCs-derived NGF, BDNF, and NT-3 were the major neuroprotectants of retinal ganglion cells (RGCs) that significantly enhanced βIII-tubulin^+^ retinal cell survival and neurite outgrowth (Mead et al., 2013). It is worth mentioning that both *in vitro* and *in vivo* experiments, DPSCs have shown greater neuroprotective and regenerative abilities than BMMSCs, which may be inseparable from their neural crest origin, indicating that DPSCs present a vital advantage over other MSCs in the nervous system repair (Ishizaka et al., 2013; Mead et al., 2014; Venugopal et al., 2022).

**FIGURE 4:**
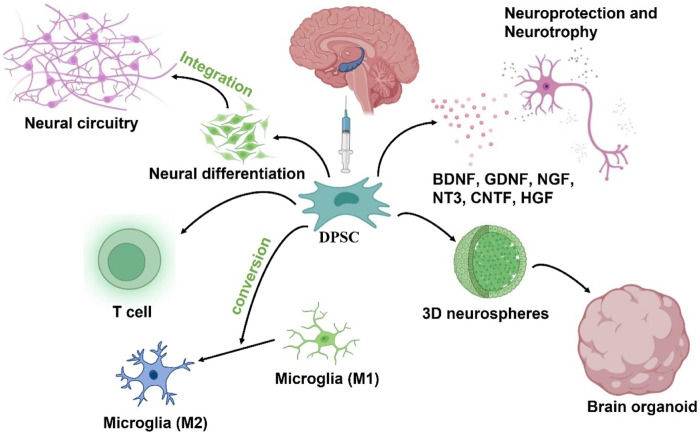
Putative DPSCs mechanism of action to treat AD: 1) Neural differentiation to replace damaged neurons, 2) Secretion of neuroprotective and neurotrophic factors, 3) neuroimmunomodulation. The engrafted DPSCs would differentiate into nerve cells to replace damaged neurons in the AD brain while integrating into existing neural circuitry. After transplantation, DPSCs could also protect neurons in the brain by secreting neuroprotective and neurotrophic factors, and also drive the polarization of macrophages toward M2 phenotype to regulate neuroinflammation in the microenvironment. In addition, DPSC can be developed into brain organoid through neural induction and 3D culture schemes for *in vitro* simulation experiments.

In summary, the therapeutic benefits of transplanted DPSCs in injured CNS might be due to paracrine effects. These neural-related factors secreted by DPSCs not only play neuroprotective and nutritional roles, but also participate in the immune regulatory response following injury in a positive way (Figure 4).

## 5 Conclusion and future prospects

Currently, AD is undoubtedly the major medical crisis facing human society. During the last several years, despite the exogenous stem cell-based therapies have gained immense progress in AD preclinical studies, it remains under further exploration. Through this review, we have described to our perspective that the neurobiology aspects of AD and the potential therapeutic prospects of DPSC according to the relevant pathological progression of AD. Indeed, although considerable progress has been made in the mechanism research and treatment exploration of AD, various preclinical studies and animal experiments based on the AD hypothesis are still difficult to translate into trials on human being. The precise microenvironment of the human brain and the complex pathophysiological generation of AD make relevant studies insufficient to support its thorough simulation, which also poses a challenge to the beneficial effects of conventional drugs in AD. Thus, many problems that need solving remain before they can be extended to clinical applications.

Given the complex pathophysiological characteristics shown in the development of AD, a multimodal treatment strategy may be required. Unlike conventional drugs with a single therapeutic target, the advantages of stem cell therapy for AD lie in the possible endogenous neurogenesis and exogenous neuronal replacement, as well as the neurotrophic and protective capacity of the secretory group. However, one of the main obstacles to utilize exogenous stem cells for AD is the selection of an appropriate cell type that are characterized by low ethical concerns, easily accessible and higher potential for nerve repairment. As an autologous stem cell considered as clinical waste, DPSCs may be the optimum cell source for exogenous stem cell therapy for AD attributed to its great advantages in neural regeneration. On the other hand, the ability of stem cells to maintain their original properties during long-term cryopreservation is also a challenge for translating stem cell therapy to clinical trials. Thus far many attempts at cryopreservation of DPSCs have obtained positive reviews. Additionally, it is noteworthy that it is only necessary to preserve unnecessary teeth without striking ethical and safety concerns.

Of course, we have to admit that many attempts to repair AD with stem cells alone have yielded only modest results, and DPSCs are unlikely to change this situation. However, with the rapid development of tissue engineering in regenerative medicine, DPSCs are apparently able to exert more effective neural differentiation and protective properties in the presence of growth factors and biomaterials. Furthermore, optimizing the properties of stem cells by genetic engineering approaches is beneficial to improve the safety, efficacy, and individualization treatment strategies of stem cell therapy. In conclusion, it seems not to be a long-distant goal soon that DPSCs in combination with biomaterials and/or nanotechnology is applicated to recoup lost neurons and integrate them into existing neural circuits and to actively restore the healthy brain microenvironment in AD.
